# Appendicitis Misdiagnosed as Peritoneal Dialysis-Related Peritonitis: A Case Report

**DOI:** 10.7759/cureus.43616

**Published:** 2023-08-17

**Authors:** Marah Khaldy, Hasan Arafat, Aseel Samoudi, Belal Shawamreh

**Affiliations:** 1 Department of Internal Medicine, Augusta Victoria Hospital, Jerusalem, PSE

**Keywords:** laparotomy, antibiotics, perforated appendix, peritoneal dialysis, peritonitis

## Abstract

Abdominal pain in patients on continuous ambulatory peritoneal dialysis (PD) is considered a serious, alarming symptom since they are at increased risk for exogenous infection introduced through the peritoneal catheter. Differential diagnosis includes perforated peptic ulcer, cholecystitis, ischemic colitis, appendicitis, diverticulitis, ingestion of foreign body, or malignancy. Typical features of perforation include signs of acute abdomen in addition to free air under the diaphragm detected on imaging. Perforation is an indication of emergency laparotomy, which is the gold standard for diagnosis.

We reported a case of a 23-year-old Palestinian male patient who had recently started continuous ambulatory PD. He presented with generalized, colicky abdominal pain associated with nausea and vomiting. He noticed turbid peritoneal fluid while performing daily dialysis sessions. The patient was diagnosed with PD-related peritonitis but had no improvement on intraperitoneal antibiotics. Further evaluation revealed an intraabdominal cause, a perforated appendix. He underwent an urgent laparotomy and switched to hemodialysis. The patient expired due to the complications of his disease.

Although PD-related peritonitis is readily diagnosed in patients on continuous ambulatory PD, an underlying intraabdominal pathology should be suspected when the condition is resistant to conventional treatment. Accepting or refuting such a diagnosis can be challenging.

## Introduction

Acute abdominal pain is a serious, alarming symptom in patients on continuous ambulatory peritoneal dialysis (CAPD). These patients are at an increased risk for exogenous infection introduced through the peritoneal catheter [1]. Peritonitis remains the most common complication of CAPD. Usually, intraperitoneal (IP) antibiotic therapy results in successful resolution of the condition. In some cases, peritonitis is resistant, and an alternate, intraabdominal, endogenous cause of peritonitis should be suspected [2]. Intestinal perforation is considered one of the important causes of endogenous peritonitis, presenting with signs of acute abdomen, such as severe abdominal pain, guarding, and rigidity. A differential diagnosis for the underlying cause of perforation includes perforated peptic ulcer, cholecystitis, ischemic colitis, appendicitis, diverticulitis, ingestion of foreign body, malignancy, or, on rare occasions, indwelling catheters. Typical features include free air in the peritoneal cavity detected on computed tomography (CT). Perforation is an indication of emergency laparotomy, which is the gold standard for diagnosis [3]. We present a case of a patient on peritoneal dialysis (PD) with perforated appendicitis resulting in endogenous peritonitis, misdiagnosed as CAPD-induced peritonitis.

## Case presentation

A 23-year-old Palestinian male patient, previously diagnosed with end-stage renal disease caused by diabetic nephropathy, was initiated on CAPD thrice daily in November, following six months of consistent hemodialysis treatment. The patient presented to the weekly nephrology clinic complaining of generalized abdominal pain, colicky in nature, nonradiating, progressive in character, and not relieved with simple analgesia. He noticed turbid color fluid during PD sessions. He had a preexisting medical history of hypertension, along with type 1 diabetes mellitus complicated by retinopathy and nephropathy. His course was complicated by many episodes of peritonitis secondary to PD. His previous surgical history included the insertion of a Tenckhoff catheter in October 2022, which was subsequently replaced in January 2023 due to recurrent *Escherichia coli* - extended-spectrum β-lactamase (ESBL) peritonitis diagnosed 10 days before the current incident. His home medications included doxazocin, aspirin, a vitamin D analog, calcium carbonate, insulin aspart, insulin detemir, and oral iron tablets. On physical examination, his blood pressure was 160/100 mmHg, temperature 36.5 °C, and heart rate 130 beats per minute. On abdominal examination, he showed diffuse abdominal tenderness with no rigidity, guarding, or rebound tenderness. Tenckhoff catheter was in situ without any sign of erythema or skin changes. He had grade +3 pitting edema reaching the middle tibia in both lower limbs. Cardiovascular and respiratory examinations were unremarkable except for the presence of tachycardia.

Investigations

**Figure 1:**
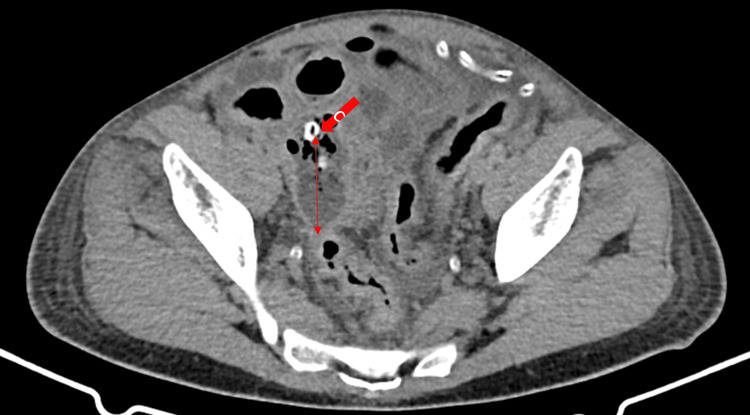
Coronal CT scan of the abdomen. Imaging shows a thick fluid collection suggestive of an appendicolith. The large arrow shows the source of obstruction, while the double-headed arrow shows the fluid collection. CT, computed tomography

Laboratory investigations showed hypokalemia (2.5 mEq/L), hyperphosphatemia (5.1 mg/dL), hypoalbuminemia (2.2 g/dL), hyponatremia (130 mmol/L), high C-reactive protein (CRP, 184 mg/L), high creatinine (8.9 mg/dL), and high blood urea nitrogen (52 mmol/L). His hemoglobin level was 11.7 g/dL and his white blood cell count (WBC) was 7.7 x 10^9^ L^-1^, of which 89% were granulocytes. Peritoneal fluid was turbid; analysis showed a WBC count of 8,040, of which 88.3% were polymorphonuclears (PMNs). The patient was diagnosed with relapsing PD-related peritonitis. Blood, urine, and peritoneal fluid culture were obtained, and the patient was started empirically on IP gentamicin according to his last peritoneal fluid culture sensitivity. Evaluation after 48 hours showed no improvement in his clinical condition, with an increase in the severity of vomiting and abdominal pain. The case was discussed with the surgical team, which recommended abdominal CT for further evaluation, suspecting an intraabdominal pathology. Imaging revealed an inflammatory process in the right lower abdomen with signs of peritonitis and abscess formation, features highly suggestive of perforated appendicitis with signs of small intestinal obstruction (Figure 1).

Treatment

The patient underwent exploratory laparotomy, revealing multiple interloop abscesses, pustule collections, a perforated appendix, and adhesional small bowel obstruction. Appendectomy, adhesiolysis, and peritoneal lavage were performed. The Tenckhoff catheter was removed, and a sample of peritoneal fluid was taken for culture. The patient was transferred to the intensive care unit for close monitoring. Blood and urine cultures returned sterile, but the peritoneal fluid culture showed the presence of *E. coli* ESBL, which was susceptible to gentamicin. IP antibiotics were discontinued, and intravenous (IV) administration of gentamicin and vancomycin was initiated. As the perm-a-cath was still in place, the patient was switched to regular hemodialysis; however, he expired within a few hours following the surgery.

## Discussion

Diagnosis of acute appendicitis in patients with PD is considered challenging. This is attributed to the fact that patients on CAPD have frequent exchanges of peritoneal fluids, in addition to a low threshold for the administration of IP antibiotics [4]. These two factors together decrease bacterial load, which delays the formation of IP abscess and consequently conceals the typical clinical signs and symptoms of acute appendicitis [1]. Direct visualization of abdominal contents is the gold standard for establishing a diagnosis of secondary peritonitis; however, the resulting disruption of abdominal content and the need for cessation of CAPD disfavor such an option. Nephrologists, on the other hand, are aware of the relative rarity of secondary peritonitis. As a result, they frequently exhibit reluctance to proceed with laparotomy unless it is considered a last resort [5].

The literature review recommends proceeding to further investigations with abdominal CT scans and even laparoscopy in nonconclusive cases [2,6]. An alternate diagnosis is highly suspected when there is a lack of clinical improvement or even worsening within the first 48 hours of antibiotic administration. Polymicrobial peritonitis with enteric organisms or anaerobes is also an indication for suspecting an alternate diagnosis, which prompts further investigations [7].

In our case, the patient lacked the characteristic clinical features of appendicitis. He had no history of localized periumbilical pain radiating to the right lower quadrant region, nor did he exhibit rebound tenderness. Due to his previous episode of PD-related peritonitis only 10 days before this episode, there was a low suspicion for an alternate diagnosis. However, the lack of clinical improvement after the administration of IP antibiotics raised the suspicion of a more serious problem, which turned out to be acute appendicitis, confirmed via CT scan and exploratory laparotomy.

## Conclusions

Endogenous causes of peritonitis are often missed in patients with CAPD. Managing patients based on wrong diagnoses can lead to significant morbidity and mortality. We recommend proceeding to alternate differential diagnosis in patients diagnosed with CAPD-induced peritonitis who lack significant improvement despite 48 hours of IP antibiotics.
